# Müller muscle conjunctival resection: Impact on Hering’s law and facial symmetry in unilateral ptosis repair

**DOI:** 10.5935/0004-2749.2024-0318

**Published:** 2025-06-24

**Authors:** Emine Savran Elibol, Nejla Tükenmez Dikmen

**Affiliations:** 1 Department of Ophthalmology, Bahçeşehir University, Istanbul, Turkey; 2 Department of Ophthalmology, Istanbul Sultan Abdulhamid Han Training and Research Hospital, Istanbul, Turkey

**Keywords:** Müller muscle conjunctiva resection, Hering’s law, Eyelids, Blepharoptosis, Reflex, Oculomotor muscles

## Abstract

**Purpose:**

Ptosis is characterized by drooping of the upper eyelid, often requiring surgical intervention for functional and aesthetic purposes. Müller’s muscle conjunctival resection is a commonly utilized surgical technique to correct mild to moderate ptosis. This retrospective study aimed to evaluate the impact of Hering’s law on the outcomes of unilateral Müller’s muscle conjunctival resection surgery, particularly eyelid and brow symmetry.

**Methods:**

Thirty patients with unilateral ptosis underwent Müller’s muscle conjunctival resection. Preand postoperative assessments included ipsilateral and contralateral side margin-reflex distance and brow position, measured through digital image analysis.

**Results:**

We found significant improvements in postoperative margin-reflex distance measurements in the ipsilateral eyelid but not in the contralateral eyelid, indicating minimal influence of Hering’s law. Brow position showed a statistically significant increase on the contralateral side but not on the ipsilateral side.

**Conclusion:**

Müller’s muscle conjunctival resection effectively restores symmetry in eyelid height and maintains brow symmetry. This is the first study to explore bilateral eyelid and brow symmetry after unilateral Müller’s muscle conjunctival resection surgery for mild to moderate ptosis. Further research should be conducted to understand the long-term effects of Müller’s muscle conjunctival resection on facial aesthetics, particularly in relation to brow position.

## INTRODUCTION

Ptosis is defined as the drooping of the upper eyelid lower than its normal anatomical location, which often requires surgical correction for functional and aesthetic purposes^(1)^. The primary elevators of the eyelid are the Müller muscle and levator palpebrae (LP), and dysfunction of these muscles leads to ptosis^(2)^. Müller’s muscle conjunctival resection (MMCR) surgery is commonly used to correct functional and aesthetic eyelid issues in cases of mild to moderate ptosis^(3)^.

Hering described the phenomenon of equal innervation for dependent extraocular muscles^(4)^. Clinically, according to Hering’s law, a patient’s effort to overcome ptosis in one eye results in increased innervation to both levators, causing contralateral eyelid retraction^(5)^.

The effect of Hering’s law has been studied in relation to different etiologies of ptosis and surgical methods^(6)^. This study examines the effects of Hering’s law on the outcomes of unilateral ptosis cases corrected using the MMCR technique.

Clinically and empirically, patients with eyelid ptosis tend to maintain a higher brow position. However, this brow elevation carries functional and aesthetic consequences, including increased forehead wrinkling, altered facial expression, and compromised facial symmetry^(7)^. In ptosis cases, increased tone of the frontalis muscle compensates for the lowered eyelid by elevating the brow and raising the eyelid margin. Surgical intervention may alleviate the need to maintain elevated brows by utilizing the frontalis muscle. Changes in brow position have been reported after ptosis surgery^(7^,^8)^. However, this is not a universal phenomenon, as brow elevation persists even after ptosis surgery in some cases^(8)^. Despite known improvements in eyelid symmetry and brow position, knowledge on the effects of MMCR on brow symmetry is limited. This study reports the outcomes on ipsilateral and contralateral upper eyelid height and brow position following unilateral MMCR surgery. To the best of our knowledge, this is the first study that investigated bilateral eyelid and brow levels and upper facial asymmetry after surgical repair of unilateral mild to moderate ptosis using MMCR. We hypothesize that brow and eyelid symmetry will be enhanced by using MMCR in patients with eyelid ptosis.

## METHODS

We conducted a retrospective review of all patients who underwent surgical repair of unilateral mild to moderate ptosis with MMCR at Dünyagöz Hospital between July 1, 2023, and July 1, 2024. We excluded those who underwent bilateral surgery, those with a postoperative follow-up period of <3 months, or those whose photographic image quality was insufficient for analysis. Patients with weak levator function (i.e., <5 mm of levator excursion), significant ocular surface disease, or a history of superior trabeculectomy were not eligible for posterior ptosis surgery with MMCR. Additionally, we excluded those who underwent simultaneous or subsequent blepharoplasty and brow surgery due to the demonstrated minimal effect of blepharoplasty on brow position. Furthermore, we also excluded patients with a history of thyroid eye disease, chronic progressive external ophthalmoplegia, myasthenia gravis, facial paralysis, or heterotropia. The Research Ethics Committee at Bahçeşehir University approved the study’s methods, and the research strictly followed the tenets of the Declaration of Helsinki.

Preoperative evaluation included a comprehensive slit-lamp examination, visual acuity testing, and assessment of ptosis. Medical and general histories were obtained. Photographs of the patients were taken preoperatively and 3 month postoperatively. The ptosis examination comprised measurements of the upper eyelid margin-reflex distance (MRD1), levator function (LF), palpebral fissure width, and the presence of Bell phenomenon. Ptosis is traditionally classified as mild (2 mm), moderate (3 mm), or severe (4 mm). We only included cases with mild and moderate ptosis for surgical intervention.

Hering’s dependence was assessed using manual elevation of the ptotic eyelid to the desired position with a single finger during the initial evaluation, followed by a reassessment of the contralateral margin-reflex distance (MRD1). Additionally, the patients considered for the MMCR procedure underwent a 2.5% phenylephrine test and manual elevation test, which aids in determining the effect of Hering’s law. The test involves instilling two drops of a 2.5% phenylephrine solution into the eye. MRD1 was re-evaluated after 5 min. An increase of ≥2 mm in MRD1 is considered a positive test. Patients exhibiting no descent of the contralateral eyelid during the phenylephrine test and responding positively to the test underwent unilateral MMCR procedures.

Patients’ photographs were taken in a seated position, with their eyes in primary gaze both preoperatively and at each follow-up visit. All measurements were performed with the patients’ heads in a neutral position, ensuring no lateral rotation, and the Frankfort horizontal plane was kept parallel to the ground to maintain consistent pupil alignment. To ensure standardization, a single researcher (E.S.E.) reviewed all patient photographs, selected the most representative images, and conducted digital analysis using NIH ImageJ software version 1.48. To ensure image quality and accuracy, a second researcher (N.T.D.) also evaluated the selected photographs. Because the measurements were derived through image analysis, the prefix “i” was used to denote “image” for measurements specific to this study. The prefix “i” was applied when discussing general terms. As described below, the evaluated outcome measurements included both the image marginal reflex distance (iMRD1) and image brow position (iBP) for the operated and nonoperated sides.

Measurements were performed using ImageJ software (National Institutes of Health, Bethesda, MD, USA). Calibration for pixel-to-millimeter conversion was performed using the average horizontal width of the cornea measured from limbus-to-limbus on each photograph as the standard reference. Specifically, the corneal diameter was marked to obtain a measurement scale, with a value of 11.7 mm assigned based on normative data from patients aged 10-80 years old.

**Figure 1:**
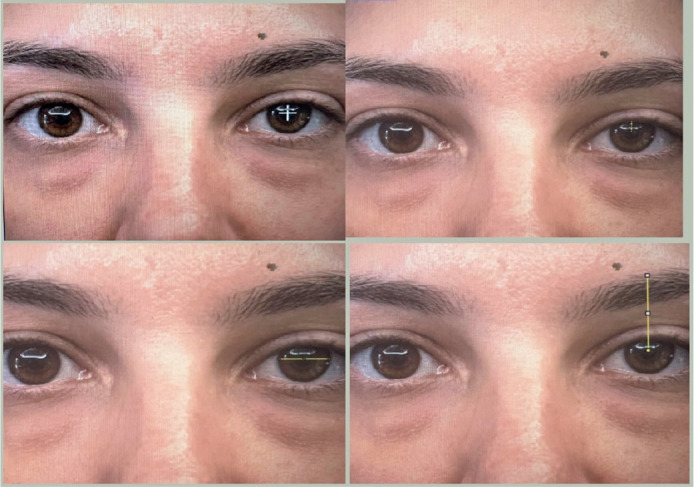
Image analysis technique. To obtain a measurement scale, the corneal diameter was demarcated and assigned a value of 11.7 mm. The pupil centroid was determined using the digital measurements of the distance from the pupil centroid to the upper eyelid margin (iMRD1) and from the pupil centroid to the superior limit of the eyebrow (iBP) using the high-zoom function. iBP, image brow position; iMRD1, image marginal reflex distance.

Digital measurements from the pupil centroid to the upper eyelid margin (iMRD1) and from the pupil center to the upper border of the eyebrow (iBP) were obtained along the midpupillary line using the high-zoom function (Figure 1). Mean changes in iMRD1 and iBP values were calculated for operated and nonoperated sides preand postoperatively using standard techniques. Additionally, preand postoperative upper eyelid asymmetry was determined through standard calculations.

### Statistical analysis

The study findings were evaluated using statistical analyses conducted with Statistical Package for the Social Sciences 26 (SPSS 26). Quantitative variables were presented with descriptive statistical methods, including mean, standard deviation, median, minimum, and maximum values, whereas qualitative variables were displayed as frequency and percentage. The Shapiro-Wilk test and box plot graphs were used to assess normality of data distribution. The Student’s t-test was used for comparisons of quantitative variables with normal distribution between groups, whereas the paired sample t-test was applied for within-group evaluations. The Mann-Whitney U test was utilized to assess nonnormally distributed variables between two groups. Fisher’s exact test was used to compare qualitative data. The results were evaluated at a significance level of p<0.05, within a 95% confidence interval.

## RESULTS

We included 30 cases, comprising 60 eyes, with 40% (n=12) male and 60% (n=18) female participants. Of the 30 cases, 30% (n=9) and 70% (n=21) underwent the procedure on their right and left eyes, respectively.

Preoperative iMRD1 measurements in the contralateral group were statistically significantly higher than those in the ipsilateral group (p=0.001; p<0.01) (Table 1).

**Table 1:** Comparison of changes in ıpsilateral and contralateral iMRD1 and iBP (mm)

	Preoperative iMRD1 Mean ± SD Min-Max	Postoperative iMRD1 Mean ± SD Min-Max	p-value	Preoperative iBP Mean ± SD Min-Max	Postoperative iBP Mean ± SD Min-Max	p-value
Ipsilateral	2.31 ± 0.76 2.3 (0.5-3.6)	3.61 ± 0.65 3.6 (1.7-4.9)	0.001^**^	21.26 ± 2.98 21.3 (15.3-27.5)	21.80 ± 3.10 22 (16.4-28)	0.083
Contralateral	3.81 ± 0.77 3.8 (2.4-5.1)	3.85 ± 0.65 3.9 (2.2-5.1)	0.688	20.43 ± 2.96 21 (14.9-25.7)	21.31 ± 3.17 21.4 (16.4-28.4)	0.006^**^
p-value	0.001^**^	0.156		0.283	0.552	

a Paired samples t-test

Student’s t-test

** p<0.01

** p<0.01.

SD= Standard deviation; Min= minimum; Max= maximum; iMRD1= image margin-reflex reflex distance; iBP= image brow position; mm= millimeter.

Postoperative iMRD1 measurements showed no statistically significant difference between the groups (p>0.05) (Table 1).

Although the preoperative ptosis group had a higher iBP than the nonptotic side, the preand postoperative iBP measurements showed no statistically significant difference between the ipsilateral and contralateral sides (p>0.05) (Table 1).

**Figure 2:**
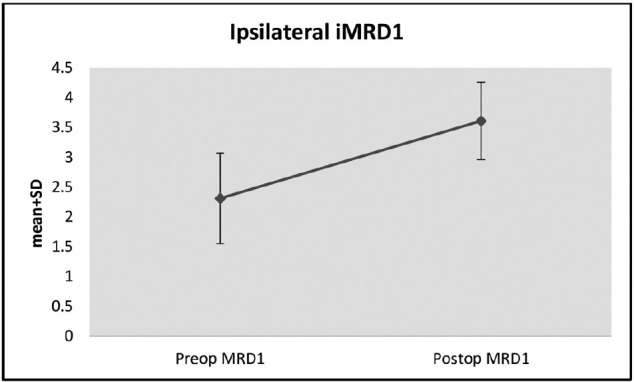
Changes in ipsilateral iMRD1 measurements preand postoperatively.

The average increase of 1.29 ± 0.51 units in postoperative ipsilateral MRD1 measurements compared with preoperative values was statistically significant (p=0.001; p<0.01) (Table 1 and Figure 2).

**Figure 3:**
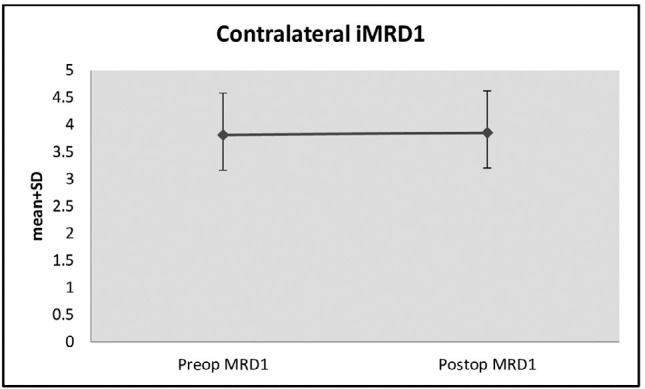
Changes in contralateral iMRD1 measurements preand postoperatively.

The changes in postoperative contralateral iMRD1 measurements compared with preoperative values were statistically significant (p>0.05) (Table 1 and Figure 3).

**Figure 4:**
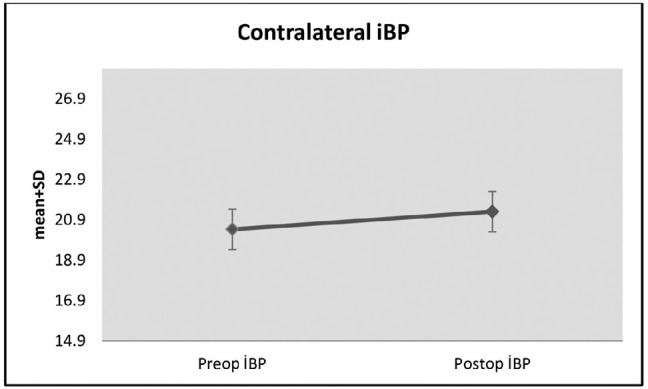
Changes in contralateral iBP measurements preand postoperatively.

Changes in ipsilateral iBP measurements preand postoperatively were not statistically significant (p>0.05). However, the average increase of 0.88 ± 1.61 units in contralateral iBP measurements postoperatively compared with that preoperatively was statistically significant (p=0.006; p<0.01) (Table 1 and Figure 4).

**Figure 5:**
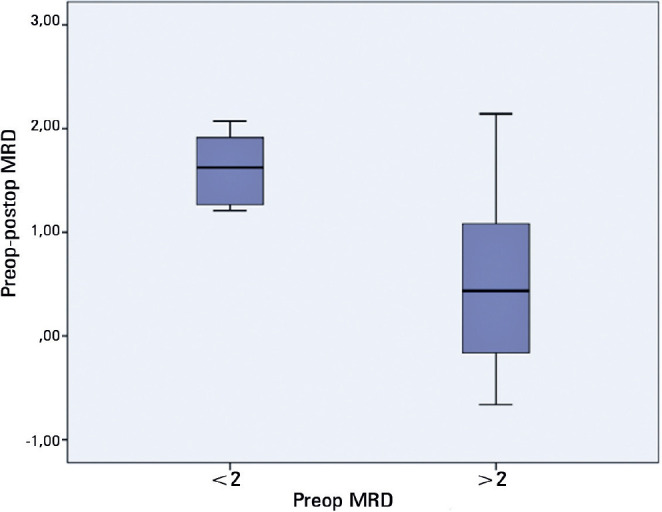
Distribution of ipsilateral preand postoperative iMRD1 changes in mild and moderate ptosis cases.

When the preoperative degrees of ptosis were categorized into moderate (iMRD1 <2 mm) and mild (iMRD1 ≥2 mm), the change in iMRD1 in moderate ptosis cases was statistically significantly higher than mild ptosis cases (p=0.022; p<0.05) (Table 2 and Figure 5).

**Table 2:** Comparison of iMRD1 and iBP changes according to ptosis degrees

		Ipsilateral Preop iMRD1(mm)		^b^p
		<2 (n=9)	>2 (n=21)	
**Preop-Postop iMRD1** **variation (Δ)**	Mean ± SD	1.62 ± 0.37	1.16 ± 0.51	0.022^*^
	Min-Max	1.62 (1.21-2.07)	1.07 (0.51-2.14)	
**Preop-Postop iBP variation (Δ)**	Mean ± SD	0.84 ± 1.58	0.40 ± 1.67	0.483
	Min-Max	0.62 (-1.8-2.85)	0.47 (-2.53-3.61)	

b Mann-Whitney U Test

* p<0.05.

SD= Standard deviation; Min= minimum; Max= maximum; iMRD1= image margin-reflex reflex distance; iBP= image brow position; mm= millimeter.

Although the change in ipsilateral iBP height in the moderate ptosis group was higher than in the mild ptosis group, the postoperative change rates were similar (p=0.483; p>0.05). When the ipsilateral preoperative iMRD1 values were classified based on a cutoff of 2, no statistically significant difference was found between the postoperative iBP difference values (Table 2).

## DISCUSSION

The significant difference in preoperative ipsilateral and contralateral iMRD1 measurements together with the lack of difference between ipsilateral and contralateral measurements postoperatively underscores the success of the MMCR procedure. Additionally, the desired symmetry in both eyelids was achieved postoperatively, evidenced by the absence of differences between postoperative ipsilateral and contralateral iMRD1 values. Studies examining this technique and its modifications reported high rates of postoperative eyelid symmetry with low complication rates^(9)^.

Walsh Hering is the first person to define the law for eyelids, describing the compensatory retraction of the contralateral upper eyelid in unilateral ptosis cases^(10)^.

The development of ptosis in the contralateral upper eyelid after successful repair of unilateral ptosis has been reported in 9.6%-17% of patients^(5^,^11)^.

Contralateral eyelid retraction is influenced by several factors, including the frontalis muscle, orbicularis oculi, sympathetic reactions from the Müller muscle, and the presence of mild ptosis in the opposite eye. Additionally, compensatory retraction may not be observed if both eyes were used for fixation^(12)^. Moreover, ocular dominance likely has a significant impact on surgical outcomes^(5)^. Our study showed that the lack of difference between preand postoperative contralateral iMRD1 values indicates that Hering’s law does not significantly affect our clinical outcomes.

Another unanswered question is whether Hering’s law manifests similarly when different surgical approaches are used. The effect of Hering’s law was examined in 52 patients with unilateral ptosis undergoing either MMCR or levator aponeurosis advancement surgery. The results showed that 19% and 52% of the cases that underwent MMCR and levator advancement surgery, respectively, required intervention on both eyelids. This study reported that the influence of Hering’s law was more prevalent in the levator advancement approach compared with MMCR^(13)^.

Levator advancement with anterior repair and MMCR with posterior repair are two entirely distinct techniques. In MMCR, the Müller muscle regulates the upper eyelid position. However, this mechanism is bypassed in cases where the levator aponeurosis is directly sutured to the tarsal plate. The levator aponeurosis influences the eyelid level instead of the Müller muscle^(14)^. After MMCR, the muscle length does not affect levator activity because the length decreases, but muscle tension does not diminish. Therefore, the effect of Hering’s law in MMCR is less pronounced than in levator advancement, as muscle tension is a more significant determinant in developing the Hering effect. Consequently, tension is absent on the nucleus of the LP (which is innervated bilaterally) or on the LP of the other eye^(15)^.

A study showed that a significant percentage of patients with congenital ptosis exhibited the Hering effect clinically, regardless of the surgical method used, even if it was not statistically significant^(16)^. Conversely, another study found that the Hering effect was not observed in cases with weak LF, as well as in post-traumatic and congenital ptosis cases (even those with good LF)^(13)^.

In the preoperative assessment, patients who responded to the phenylephrine test and underwent unilateral Müller’s muscle resection were those where contralateral ptosis did not develop due to the Hering effect. Patients who developed ptosis in the contralateral eyelid after the phenylephrine test underwent bilateral MMCR and were excluded. Consequently, the absence of Hering dependence in patients who underwent unilateral MMCR may be attributed to the failure to observe this effect in the preoperative evaluation. Additionally, MMCR is not an appropriate method for patients with severe ptosis, as only a maximum of 2 mm resection can be elevated by posterior lamellar shortening. The lack of the Hering effect in patients with mild to moderate ptosis may be due to lower levator muscle tension.

When classifying the surgical patients based on moderate and mild MRD1, the preand postoperative iMRD1 differences in eyes with moderate ptosis were greater than those with mild ptosis, indicating that eyelids with more severe ptosis had a greater elevation and symmetry restoration effort following MMCR. This phenomenon could be related to the more significant excision of the Müller muscle in more severe ptosis cases, or it may reflect the ability of intact Müller muscle to balance the eyelids physiologically. The absence of the Hering effect might also be attributed to this balancing mechanism.

The physiological mechanisms underlying eyebrow asymmetry during ptosis are not fully understood. Eyebrow asymmetry is rare in normal individuals^(17)^. However, eyebrow height asymmetry is observed in approximately 25% of individuals with ptosis^(18)^. Ptosis surgery also affects eyebrow position, generally resulting in a clear downward effect^(7^,^19)^. The average iMRD1 of 2.31 on the preoperative surgical side indicates mild to moderate ptosis in patients undergoing MMCR. Consequently, we may not have observed a significant difference in preoperative ipsilateral and contralateral brow heights. Typically, compensatory brow elevation occurs in cases of more severe ptosis. The lack of difference in brow height preand postoperatively on the affected side supports this hypothesis, despite ptosis resolution. However, the average increase of 0.88 ± 1.61 units in postoperative contralateral iBP measurements, considering the absence of changes on the ipsilateral side, is unexpected. Hering’s law of equal innervation also applies to asymmetric brow ptosis, indicating a change in brow height may be due to eyelid correction on the contralateral side. Such interactions could be considered as side effects of the changes in eyelid position and may facilitate a better understanding of aesthetic outcomes following surgery^(11)^. Hering’s law also applies to the frontal branch of the facial nerve, resulting in the depression of the ipsilateral brow and acute involuntary elevation of the contralateral brow^(20)^. The reflex contraction of the frontalis muscle has been suggested to be initiated by tension in the eyelid retractors. In this case, prolonged eyelid position leads to reflex contraction of the frontalis muscle^(21)^. Thus, the frontalis muscle manages to maintain some degree of symmetry preand postoperatively in mild ptosis cases.

Zheng et al. described eyebrow changes following levator aponeurosis advancement in 120 eyelids and reported that the eyebrow at the center of the pupil dropped by 3.45 mm 2 months postoperatively^(22)^. Moore et al. observed a decrease of 0.83 mm in the eyebrow position at the center in 274 eyelids that underwent MMCR over a postoperative period ranging from 1 to 12 months, with a mean follow-up duration of 3.8 months^(17)^. Rootman et al. performed MMCR on 125 eyelids and reported that the eyebrow position at the center had a reduction of 1.0 mm at an average follow-up of 3.2 months, with assessments conducted approximately 1.5 months postoperatively^(7)^. Following surgical repair of ptosis targeting the Müller muscle, the eyebrows tend to descend less compared with surgical techniques that manipulate the levator aponeurosis.

Kokubo et al. assessed eyebrow heights in ptosis patients postoperatively and found that more severe instances of blepharoptosis were linked to a greater postoperative eyebrow lowering^(23)^. Blepharoptosis surgery significantly reduced eyebrow height, and the preoperative eyebrow position is the most crucial factor in predicting this change^(24)^. Another study involving 228 patients reported that 50% of those with preoperative eyebrow asymmetry had persistent eyebrwo asymmetry after undergoing MMCR ptosis surgery^(25)^. Even if MRD1 symmetry is achieved, many patients with preoperative eyebrow asymmetry will continue to experience this condition postoperatively. The inconsistency between MRD1 changes and eyebrow symmetry reflects the variability and unpredictability of the eyebrow position response to eyelid surgery.

In this study, the median postoperative follow-up period was 3 months, which may be considered arbitrary. Another study noted that nearly half of the patients who underwent MMCR experienced changes in eyelid position between the 1-week visit and the “late” follow-up visit (ranging from 12 to 52 weeks)^(26)^. However, no existing data indicate that eyelid position continues to change beyond this time frame nor any evidence suggesting that eyelid or brow symmetry becomes static at any point.

In this study, brow changes were not significantly different when comparing moderate and mild ptosis as subgroups. We can conclude that the brow response related to the Hering effect does not manifest in mild and moderate ptosis cases, similar to the findings regarding eyelid position.

However, this study has several limitations. Ptosis severity, effects of ocular dominance, and known daily fluctuations in eyelid position that play a role in changes in brow position during ptosis have not been considered. Our methodology is limited by the constraints of photographic image analysis. To establish a measurement scale, a fixed limbus-to-limbus distance of 11.7 mm was determined. Considering the variations in corneal diameter across different age groups and the normal individual differences in corneal size, the 11.7-mm scale distance may restrict the accuracy of the measurements. The study also has a relatively small sample size.

The current study indicates that Hering’s law has minimal impact on the outcomes of cases with unilateral mild to moderate ptosis. These cases can be addressed unilaterally, eliminating the need for correcting the contralateral eyelid. The preoperative assessment of the Hering effect is considered highly reliable and accurately reflects the true outcome. MMCR surgery seems to be a dependable method to achieve symmetry in eyelid positioning and maintaining symmetry in brow position.
